# Acute Yellow Atrophy of the Liver

**Published:** 1907-11-02

**Authors:** 


### ACUTE YELLOW ATROPHY OF THE LIVER.

The following is a fairly typical case of acute yellow atrophy of the liver, exemplifying the comparatively long duration and mild degree of the initial jaundice, and the conversely severe delirium of the terminal stage.

An unmarried woman, aged 28, a barmaid by occupation, had led an irregular life, not only drinking more alcohol than was good for her, but also suffering from secondary syphilis. There had been no pregnancy. One December she became slightly jaundiced, and consulted a doctor about this. No enlargement of the liver or spleen could be detected, and there had been no hæmatemesis, though there had been a good deal of retching and epigastric pain; cirrhosis of the liver seemed unlikely, notwithstanding the habits; the pains had not been severe enough to suggest gall-stones; the diagnosis made was gastro-duodenitis with catarrhal jaundice. The latter did not become very deep, though it did not actually clear up. It persisted for many weeks, sometimes being scarcely noticeable, sometimes deepening into a definite yellowness, especially after meals. This went on during January, February, and March, the illness of the patient not appearing to be very severe. The liver dullness was natural during this time.

Towards the end of April the patient almost suddenly became collapsed and semi-comatose; simultaneously there was repeated and profuse epistaxis and persistent vomiting, the vomits containing small quantities of blood that may or may not have passed down the œsophagus from the nose. The temperature rose to 101·2° F. next day, but thereafter it was subnormal, and remained between 95° F. and 96° F. until the end. It was also noted that the jaundice was deepening and that the liver dullness was rapidly diminishing. The urine was very dark with bile pigments, and tyrosine crystals were found in the urinary deposit.

After the initial adynamic period came one of violent delirium, in which the patient was constantly tossing herself about and shrieking or calling out every moment. This state of things lasted for six days, during which there were repeated attacks of epistaxis, acute delirium, tachycardia, and hypothermia. There was incontinence of urine and fæces; the pulse was small, irregular, and rapid; just before the end a particularly severe epistaxis with abundant hæmatemesis ensued, followed by deep coma which very soon terminated in death.

At the autopsy there were four pints of deeply bile-stained ascitic fluid in the peritoneal cavity; the stomach and colon were acutely dilated with gas. The kidneys were healthy; the spleen was slightly enlarged, weighing six ounces; the heart was flabby, but otherwise natural; the lungs were congested and œdematous; the brain was normal; the main morbid changes were in the liver. This organ weighed only twenty-six ounces, and it was so soft that it flopped into any shape. The gall bladder was moderately distended. The liver substance showed the typical bold mottling, some areas being almost bright orange, others pale yellow, and others, again, dull reddish-brown. The different areas were very distinctly marked out, some of them looking almost like adenomata amid the rest of the liver substance. Microscopical sections of the organ were with difficulty recognisable as hepatic; for the most part the liver cells had undergone granular disintegration, but here and there some foci showed the remains of parenchyma with intracellular fat globules. There was no evidence of acute inflammation in the sense of small round-celled infiltration, and there was no cirrhotic fibrosis. The main changes in the liver were less those of a fatty nature than those of an acute granular necrosis, and this, with the marked diminution in the size and weight of the organ, is typical of what is usually seen in acute yellow atrophy.

